# CD71^+^ Erythroid Cells Exacerbate HIV-1 Susceptibility, Mediate *trans*-Infection, and Harbor Infective Viral Particles

**DOI:** 10.1128/mBio.02767-19

**Published:** 2019-11-26

**Authors:** Afshin Namdar, Garett Dunsmore, Shima Shahbaz, Petya Koleva, Lai Xu, Juan Jovel, Stan Houston, Shokrollah Elahi

**Affiliations:** a Department of Dentistry, Faculty of Medicine and Dentistry, University of Alberta, Edmonton, Alberta, Canada; b Department of Medical Microbiology and Immunology, Faculty of Medicine and Dentistry, University of Alberta, Edmonton, Alberta, Canada; c The Applied Genomics Core, Office of Research, University of Alberta, Edmonton, Alberta, Canada; d Department of Medicine, Division of Infectious Disease, University of Alberta, Edmonton, Alberta, Canada; e Department of Oncology, Faculty of Medicine and Dentistry, University of Alberta, Edmonton, Alberta, Canada; f Li Ka Shing Institute of Virology, University of Alberta, Edmonton, Alberta, Canada; University of KwaZulu-Natal

**Keywords:** HIV, CD71^+^ erythroid cells, *trans*-infection, ROS, RBCs, CD235a, HIV transmission, human immunodeficiency virus, reservoir

## Abstract

CD71^+^ erythroid cells (CECs) have a wide range of immunomodulatory properties. Here, we show that CECs are expanded in the peripheral blood of HIV patients, with a positive correlation between their frequency and the plasma viral load. CECs from HIV patients and human cord blood/placenta exacerbate HIV-1 infection/replication when cocultured with CD4^+^ T cells, and that preexposure of CD4^+^ T cells to CECs enhances their permissibility to HIV infection. However, mature red blood cells (RBCs) do not enhance HIV replication when cocultured with CD4^+^ T cells. We also found CECs express substantial levels of the NOX2 gene and via a mitochondrial reactive oxygen species (ROS)-dependent mechanism possibly upregulate NF-κB in CD4^+^ T cells once cocultured, which affects the cell cycle machinery to facilitate HIV-1 replication. The complement receptor-1 (CD35) and the Duffy antigen receptor for chemokines (DARC) as potential HIV target molecules are expressed significantly higher on CECs compared to mature red blood cells. Blocking CD35 or DARC substantially abolishes HIV-1 transmission by RBCs to uninfected CD4^+^ T cells but not by CECs. In contrast, we observed CECs bind to HIV-1 via CD235a and subsequently transfer the virus to uninfected CD4^+^ T cells, which can be partially blocked by the anti-CD235a antibody. More importantly, we found that CECs from HIV-infected individuals in the presence of antiretroviral therapy harbor infective viral particles, which mediate HIV-1 *trans*-infection of CD4^+^ T cells. Therefore, our findings provide a novel insight into the role of CECs in HIV pathogenesis as potential contributing cells in viral persistence and transmission.

## Abstract (precis)

Immature red blood cells (erythroid precursors or CD71^+^ erythroid cells) have a wide range of immunomodulatory properties. In this study, we found that these erythroid precursors are abundant in the human cord blood/placental tissues, in the blood of HIV-infected and anemic individuals. We observed that these cells exacerbate HIV-1 replication/infection in target cells and even make HIV target cells more permissible to HIV infection. In addition, we found that HIV gets a free ride by binding on the surface of these cells and thus can travel to different parts of the body. In agreement, we noticed a positive correlation between the plasma viral load and the frequency of these cells in HIV patients. More importantly, we observed that infective HIV particles reside inside these erythroid precursors but not mature red blood cells. Therefore, these cells by harboring HIV can play an important role in HIV pathogenesis.

## INTRODUCTION

Red blood cells (RBCs) are the most abundant cells in humans (20 to 30 trillion) and normally are generated in the bone marrow (1). Traditionally, the main function of these cells has been considered as gas transporters (O_2_ and CO_2_) and preservation of systemic acid/base equilibrium (2). However, in recent years mounting evidence indicate a direct and important role for these cells in innate immunity and inflammation (3, 4). In addition to mature RBCs, a wide range of immunomodulatory functions have been assigned to their immature counterparts (5–8). One important immunomodulatory feature of human RBCs is their tendency to bind to a wide range of chemokines. The Duffy antigen receptor for chemokines (DARC) is an important binding locus, which acts as a sink for interleukin-8 (IL-8) and interacts with other chemokines (3, 4). The antioxidant property of RBCs is another major immunomodulatory role for these cells, which is essential for their function and integrity. In general, reactive oxygen species (ROS) released by neutrophils and macrophages are taken up by RBCs and get neutralized by the cytosolic antioxidant system (9–11). However, recent studies indicate that RBCs themselves also generate endogenous ROS (9–11), and interestingly their immature counterparts mediate immunomodulatory functions via the generation of ROS (12, 13). Immature erythroid cells are typically seen in the periphery of fetuses and newborns, while they are absent or in low frequency in the blood of healthy adults (3, 5). It is worth noting that under certain physiological or pathological conditions, erythroid precursors can be seen in adults (13–15). For instance, conditions such as anemia, pregnancy, chronic infection, and late-stage cancer result in extramedullary erythropoiesis and subsequently abundance of erythroid precursors in the periphery (6, 12, 13, 16).

Recently, we discovered that erythroid precursors (CD71^+^ erythroid cells [CECs]) are present in impressively high numbers in neonatal mice spleens and human cord blood. They coexpress the transferrin receptor (CD71) and the erythroid lineage marker (TER119) in mice but CD71 and CD235a in humans (17). Additionally, we found that CECs are abundant in the placental tissues and expand in the peripheral blood of pregnant women (12, 14). We and others have shown CECs have a wide range of immunomodulatory functions and suppress both the innate and adaptive immune responses (6–8, 13, 17). In light of previous reports that HIV-1 through DARC and complement receptor-1 CR1 (CD35) binds to RBCs (18), we speculated that CECs may also express CD35 and DARC.

HIV pathogenesis in early life, when CECs are physiologically abundant, is defined by rapid CD4^+^ T cell decline, higher plasma RNA levels, and accelerated progression to AIDS and death compared to adults (19, 20). Often these infections occur via mother-to-child transmission (MTCT) *in utero*, intrapartum, or postpartum (breastfeeding) (21).

Anemia is another important factor that may influence HIV pathogenesis. It is associated with the abundance of CECs in the peripheral blood. Although the role of anemia in HIV pathogenesis is complex and multifactorial (22, 23), it is a common feature of HIV-related disease and has been uniformly demonstrated to be an independent predictor of morbidity and mortality (23, 24). In addition, anemia can be caused by other pathogens, such as malaria, hookworms, or micronutrient deficiencies (23, 24). Therefore, due to the physiological expansion of CECs in the periphery of newborns, pregnant mothers, anemic individuals, and patients with other pathological conditions (e.g., cancer), it is critical to elucidate their potential effects on HIV transmission and infection/replication.

Here, for the very first time, we show that CECs regardless of source (cord blood, placenta, or peripheral blood of anemic and HIV patients) mediate the exacerbation of HIV-1 replication in CD4^+^ T cells and HIV *trans*-infection of CD4^+^ T cells. Our observations coupled with transcriptome sequencing (RNA-seq) data demonstrate how interactions of CECs with CD4^+^ T cells via ROS affect the cell cycle machinery to facilitate HIV-1 replication. In addition, we demonstrate that CECs compared to mature RBCs express significantly higher levels of both CD35 and DARC. However, our data indicate that unlike RBCs, these chemokine receptors do not play an important role in HIV *trans*-infection by CECs of uninfected CD4^+^ T cells *in vitro*. In contrast, HIV-1 binds to CD235a, and thus CECs cannot only *trans*-infect uninfected CD4^+^ T cells but also harbor infective virions in the presence of antiretroviral therapy (ART) drugs.

## RESULTS

### Increased frequency of CECs in the peripheral blood of HIV-1-infected and anemic individuals.

**FIG 1:**
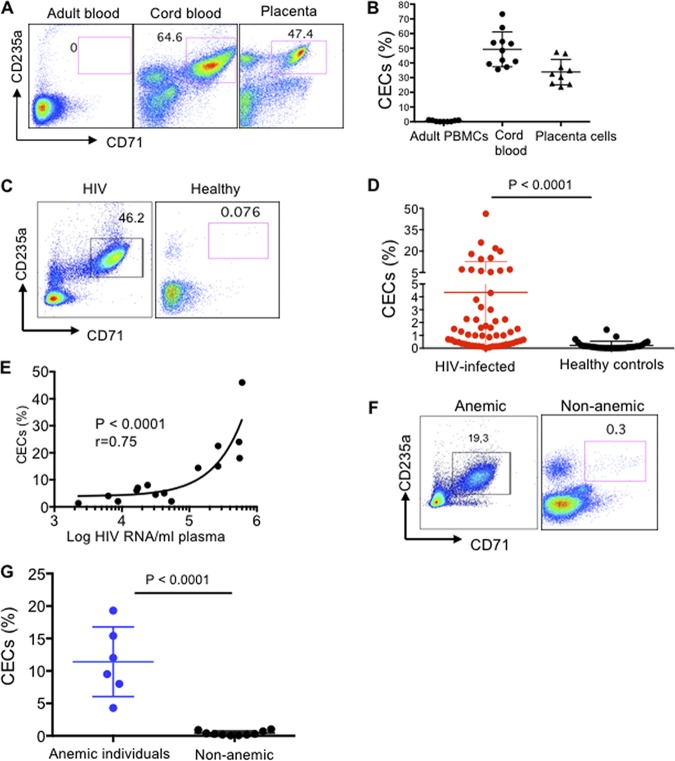
CECs are abundant in the peripheral blood of HIV-1-infected and anemic individuals. (A) Representative plots showing the frequency of CECs in adult peripheral blood mononuclear cells (PBMCs) versus the cord blood mononuclear cells (CBMCs) and placental tissue. (B) Cumulative data comparing the percentages of CECs in adult PBMCs versus CBMCs and placental tissues. (C) Representative flow cytometry plots showing the frequency of CECs in an HIV-infected individual versus healthy control. (D) Cumulative data indicating percentage of CECs in HIV-infected versus healthy individuals. (E) Data showing the correlation of CECs with the plasma viral load in HIV-infected but ART-naive individuals. (F) Representative flow cytometry plots showing frequency of CECs in an anemic versus nonanemic individual. (G) Cumulative data comparing the percentages of CECs in adult anemic versus healthy individuals.

In agreement with our previous reports (6, 17), here we show that CECs are physiologically abundant in the human umbilical cord blood and placental tissues, while they are almost absent in the peripheral blood of healthy adults (Fig. 1A and B). Interestingly, we found that CECs become abundant in the peripheral blood of HIV-infected individuals versus healthy controls (Fig. 1C and D). More importantly, we observed a positive correlation between the frequency of CECs with the plasma viral load in HIV-infected and ART-naive patients (Fig. 1E). Similarly, we observed expansion of CECs in the peripheral blood of anemic individuals (Fig. 1F and G).

### The cord blood and placental CECs but not RBCs exacerbate HIV infection in already infected autologous CD4^+^ T cells.

**FIG 2:**
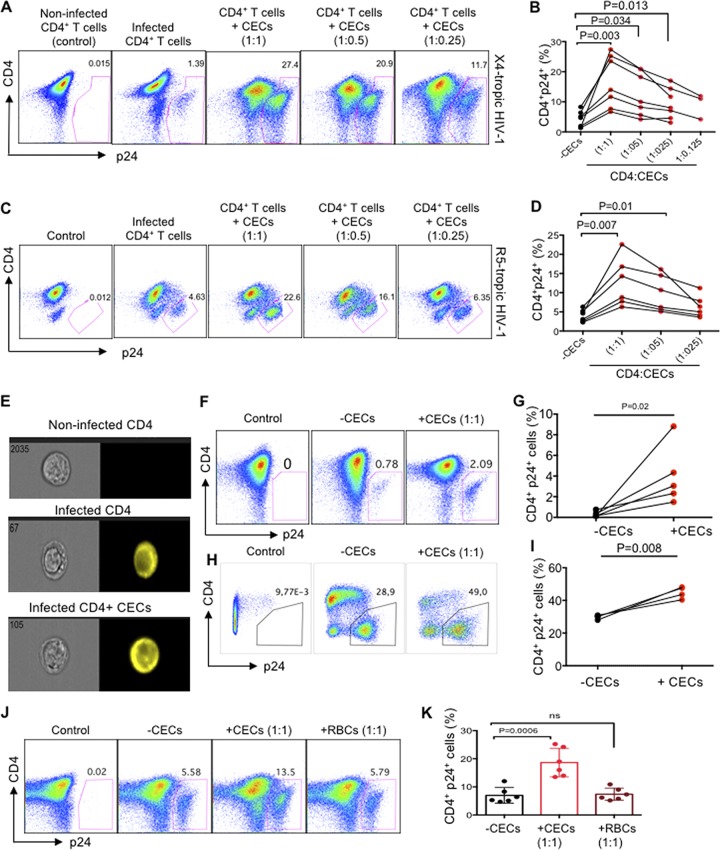
The cord blood and placenta CECs exacerbate HIV infection in already infected autologous CD4^+^ T cells. (A) Representative flow cytometry plots showing activated CD4^+^ T cells from the cord blood following infection with the X4-tropic (HIV-1_LAI_) isolate in the absence or presence of CECs. (B) Cumulative data showing percentages of activated CD4^+^ T cells after infection with the X4-tropic isolate and cocultured with CECs at different ratios. (C) Representative flow cytometry plots showing activated CD4^+^ T cells from cord blood following infection with the R5-tropic (HIV-1_JR-CSF_) isolate in the absence or presence of CECs. (D) Cumulative data showing percentages of activated CD4^+^ T cells after infection with the R5-tropic isolate in the presence or absence of CECs at different ratios. (E) Representative images showing intensity of p24 in CD4^+^ T cells in the absence or presence of CECs. (F) Representative flow cytometry plots showing inactivated CD4^+^ T cells from cord blood following infection with the X4-tropic isolate in the absence or presence of CECs. (G) Cumulative data showing percentages of nonactivated CD4^+^ T cells after infection with the X4-tropic isolate in the absence or presence of CECs. (H) Representative flow cytometry plots showing activated CD4^+^ T cells from the placenta following infection with the X4-tropic isolate in the absence or presence of placenta CECs. (I) Cumulative data showing percentages of activated and infected CD4^+^ T cells from the placenta with the X4-tropic viral isolate in the absence or presence of CECs. (J) Representative plots of CD4^+^ T cells alone (−CECs), in the presence of CECs (1:1 ratio) or in the presence of RBCs (1:1 ratio). (K) Cumulative data showing percentages of activated/infected CD4^+^ T cells (X4-tropic) from cord blood in the presence of CECs or RBCs. ns, not significant. The number of infected cells was quantified by intracellular viral p24 antigen staining using flow cytometry on day 4 postinfection. Each point represents a cord blood or placenta sample.

Since we observed an expansion of CECs in the peripheral blood of HIV-infected individuals (Fig. 1C and D)—in particular, a positive correlation between the frequency of CECs with the plasma viral load (Fig. 1E)—we decided to investigate how CECs influence HIV-1 infection in CD4^+^ T cells using an *ex vivo* infection assay. Therefore, we decided to answer these questions using cord blood CECs because of the feasibility and their abundance. Cord blood CD4^+^ T cells were isolated and made more permissible to HIV-1 infection by *in vitro* culture with exogenous IL-2 and phytohemagglutinin (PHA) stimulation (25). Subsequently, CD4^+^ T cells were infected with either the lab-adapted X4-tropic isolate (HIV-1_LAI_) or R5-tropic HIV-1 isolate (HIV-1_JR-CSF_). Isolated autologous CECs at different ratios were added to the infected CD4^+^ T cells following an extensive wash to remove extracellular viruses. Viral replication was analyzed by intracellular p24 staining using flow cytometry 3 to 4 days later. Using these culture conditions, we consistently observed that CECs significantly enhanced HIV infection in CD4^+^ T cells with both X4-tropic (Fig. 2A and B) and R5-tropic HIV-1 viruses (Fig. 2C and D). CEC-mediated enhanced HIV-1 infection in CD4^+^ T cells was dose dependent for both X4-tropic and R5-tropic viral isolates, respectively (Fig. 2B and D). We found that CECs not only significantly increased the number of infected CD4^+^ T cells (Fig. 2A to D), but also the number of viruses per cell was significantly greater, as shown by the intensity of p24 expression (Fig. 2E; see Fig. S1A and B in the supplemental material). Similarly, we found that the absolute number of infected CD4^+^ T cells was significantly higher in the presence of CECs (Fig. S1C). Consistent with activated CD4^+^ T cells, we found that CECs increased HIV-1 infection in nonactivated CD4^+^ T cells (Fig. 2F and G). The placenta-derived CECs, similar to the cord blood, significantly increased HIV-1 infection in autologous CD4^+^ T cells (Fig. 2H and I). Nevertheless, mature RBCs from the cord blood did not enhance HIV infection in CD4^+^ T cells (Fig. 2J and K), which indicates a differential role for CECs in HIV infection compared to their older siblings. Since nonactivated cord blood CD4^+^ T cells do not express substantial levels of CCR5 compared to CXCR4 (Fig. S1D) and CECs enhanced replication of both viral isolates, we decided using the X4-tropic viral isolate for the subsequent studies except when rCCL-5 and anti-CD35 antibody was used.

10.1128/mBio.02767-19.1 FIG S1 (A) Cumulative data showing MFI of p24 in infected-CD4^+^ T cells in the absence or presence of CECs. (B) Amnis ImageStream plots showing intensity of p24 in CD4^+^ T cells infected with HIV in the absence and presence of CECs. (C) Cumulative data showing the absolute infected CD4^+^ T cell count in the absence or presence of CECs. (D) Representative flow cytometry plot showing CXCR4 and CCR-5 expression on nonactivated CD4^+^ T cells from cord blood. Download FIG S1, JPG file, 0.08 MB. Copyright © 2019 Namdar et al. 2019 Namdar et al. This content is distributed under the terms of the Creative Commons Attribution 4.0 International license.

### Preexposure of CD4^+^ T cells to autologous cord blood CECs enhance their infectivity to HIV-1 infection.

**FIG 3:**
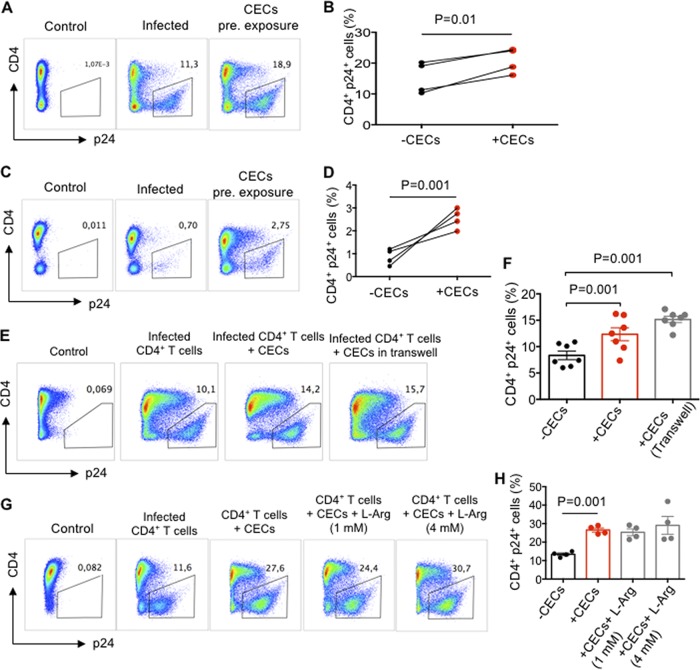
Preexposure of CD4^+^ T cells to autologous cord blood CECs makes them more permissible to HIV-1 infection. (A) Representative flow cytometry plots showing preexposed activated CD4^+^ T cells to CECs prior to infection with the X4-tropic isolate versus nonexposed activated CD4^+^ T cells to CECs. (B) Cumulative data showing percentages of p24 in activated CD4^+^ T cells either preexposed to CECs prior to infection with X4-tropic isolate or not exposed to CECs. (C) Representative flow cytometry plots showing nonactivated CD4^+^ T cells preexposed to CECs prior to infection with the X4-tropic isolate versus nonactivated CD4^+^ T cells nonexposed to CECs (D) Cumulative data showing percentages of p24 in nonactivated CD4^+^ T cells either preexposed to CECs before infection with the X4-tropic isolate or not exposed to CECs. (E) Representative flow cytometry plots showing infection with the X4-tropic isolate in activated CD4^+^ T cells cocultured with CECs versus the Transwell system. (F) Cumulative data showing percentages of CD4^+^ T cells infected with the X4-tropic isolate when cocultured with CECs versus the Transwell system. (G) Representative flow plots showing infection with X4-tropic isolate in activated CD4^+^ T cells in the presence of CECs and/or l-arginine (l-Arg) at 1 and 4 mM. (H) Cumulative data showing percentages of CD4^+^ T cells infected with HIV in the presence or absence of CECs and/or l-arginine.

Activated CD4^+^ T cells were cocultured with CECs (1:1 ratio) overnight, then CD4^+^ T cells were isolated and infected with HIV-1_LAI._ We observed that preexposure of CD4^+^ T cells to CECs significantly enhanced their infectivity to HIV-1 (Fig. 3A and B). To confirm if this was the case for nonactivated CD4^+^ T cells, similar overnight cocultures were performed. Likewise, we observed preexposure of resting CD4^+^ T cells to CECs significantly enhanced their susceptibility to HIV-1 infection (Fig. 3C and D).

### CECs via soluble mediators enhance HIV-1 infection/replication in CD4^+^ T cells.

We further decided to determine whether enhanced HIV-1 infection in CD4^+^ T cells required cell-cell interactions. By using a Transwell system, we observed that increased HIV-1 infection in CD4^+^ T cells does not necessarily depend on cell-cell interactions (Fig. 3E and F). Previously, we had demonstrated that CECs mediate immunosuppression via enzymatic activity of arginase-2 (6, 8, 14, 17). In addition, a high level of arginase activity and subsequent l-arginine deprivation has been associated with dysfunctional T cells and increased viral load in HIV patients (26). Thus, we hypothesized that arginase-2 activity by CECs may enhance HIV-1 infection/replication in CD4^+^ T cells. In contrast, we found that overriding the enzymatic activity of arginase-2 by l-arginine supplementation did not abrogate the enhanced HIV-1 infection in CD4^+^ T cells when cocultured with CECs (Fig. 3G and H). We have previously shown that CECs in mice via arginase-2 activity suppress CD11b^+^ and CD11c^+^ cells (17). To determine whether the indicated concentration of l-arginine has any biological effects on cord blood CECs, we used a bacterial phagocytosis assay in the presence/absence of CECs or l-arginine supplementation *in vitro*. As shown in Fig. S2A in the supplemental material, overriding arginase activity with l-arginine supplementation (2 mM) restored phagocytosis of cord blood CD11b cells *in vitro*. However, overriding arginase activity of CECs had no significant effects on HIV-1 infection in CD4^+^ T cells (Fig. 3G and H). Moreover, despite the fact that CECs constitutively secrete transforming growth factor β (TGF-β) (27), we found this cytokine had no effects on CEC-mediated enhanced HIV-infection/replication in CD4^+^ T cells (Fig. S2B). These data suggest that CECs—possibly by secreting other soluble factors—promote HIV-1 infection/replication.

10.1128/mBio.02767-19.2 FIG S2 (A) Representatives of 2,000 images collected using an Amnis ImageStream Mark II for each sample showing engulfed Bordetella pertussis in CD11b^+^ cells from the cord blood in the presence/absence of CECs with or without l-arginine supplementation *in vitro.* (B) Representative plots showing the percentage of p24 in CD4^+^ T cells alone or in the presence of Apo and TGF-β blocker at indicated concentrations. (C) Hierarchical clustering on Euclidian distances showing different gene expression profiles in HIV-infected CD4^+^ T cells in the presence or absence of CECs. (D) Principal-component analysis (PCA) of the Euclidian distances between HIV-infected CD4^+^ T cells in the presence or absence of CECs. Download FIG S2, JPG file, 0.09 MB. Copyright © 2019 Namdar et al. 2019 Namdar et al. This content is distributed under the terms of the Creative Commons Attribution 4.0 International license.

### CECs via oxygen-containing compounds modulate the expression of genes associated with increased CD4^+^ T cell infectivity.

**FIG 4:**
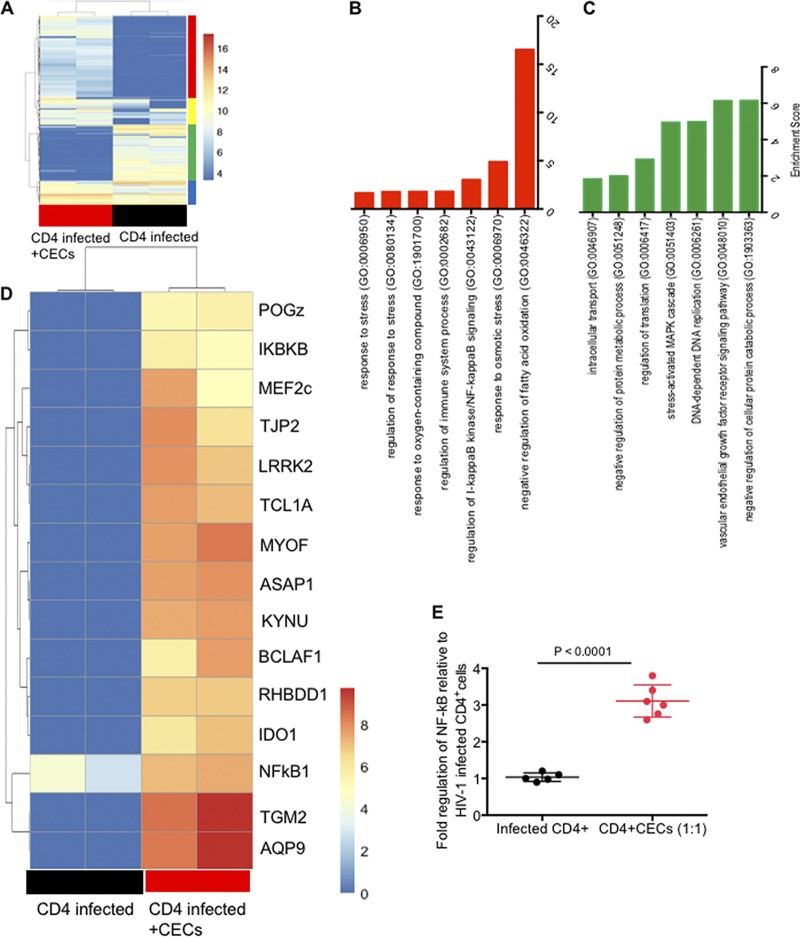
CECs via oxygen-containing compounds modulate the expression of genes associated with increased CD4^+^ T cell infectivity. (A) The transcriptome of infected CD4^+^ T cells in the absence and presence of CECs is shown using a heat map. (B) Gene Ontology analysis of the biological process for the transcriptome profile of upregulated genes in CD4^+^ T cells cocultured with CECs. (C) Gene Ontology analysis of the biological process for the transcriptome profile of downregulated genes in CD4^+^ T cells in the presence of CECs. (D) The most-upregulated genes associated with enhanced HIV infection in CD4^+^ T cells. (E) NF-κB gene expression was confirmed in CD4^+^ T cells when cocultured with CECs. Raw data have been deposited in the SRA database of NCBI and are publicly available under accession no. PRJNA529907 at https://www.ncbi.nlm.nih.gov/sra.

We implemented transcriptome sequencing (RNA-seq) to analyze the transcriptome of HIV-1-infected CD4^+^ T cells alone or when cocultured with CECs. When hierarchical clustering was conducted on Euclidian distances between samples, CD4^+^ T cells exposed to CECs clearly showed a different gene expression profile compared to CD4^+^ T cells that were not cocultured with CECs (Fig. S2C). Those results were recapitulated in principal-component analysis (PCA) on the Euclidian distances between samples (Fig. S2D). In essence, we found the transcriptome profile of infected CD4^+^ T cells cocultured with CECs was clearly separate from that of the infected CD4^+^ T cells in the absence of CECs (Fig. 4A). Gene Ontology analysis of the biological process for the transcriptome profile revealed upregulation of cellular response to oxygen-containing compounds and upregulation of NF-κB signaling in CD4^+^ cells cocultured with CECs (Fig. 4B). In contrast, negative regulation of the cellular protein metabolic/catabolic process was evident in highly downregulated genes (Fig. 4C). In total, 1,011 genes were highly upregulated (≥2-fold; false-discovery rate [FDR], ≤0.05) and 1,910 genes were downregulated (≥2-fold; FDR, ≤ 0.05) in cocultured infected CD4^+^ T cells compared to CD4^+^ T cells alone. We selected approximately 80 highly upregulated and downregulated genes (see Fig. S3A in the supplemental material). The biological functions of most of these genes in regard to HIV-1 replication/T cell proliferation are unknown. However, among these, we identified 16 highly upregulated genes in CD4^+^ T cells when cocultured with CECs (Fig. 4D), which can be associated with CEC-mediated enhanced HIV-1 infection. The tissue transglutaminase (tTg) gene was the most upregulated gene (>12-fold) followed by the AQP9 (aquaporin 9) gene. Aquaporins (AQPs [>12-fold]) are channel proteins widely present in living cells to facilitate the transport of water and certain neutral solutes across biological membranes. The third highly upregulated gene was MYOF (>10-fold), which is a membrane-associated protein involved in both caveolin and clathrin-mediated endocytosis pathways along with membrane fusion after damage (28). It has been reported that upregulation of AQP9 is required as ROS scavenger to prevent T cell apoptosis (29). Other highly upregulated genes were ASAP1 (ArfGAP with SH3 domain, ankyrin repeat, and PH domain 1), KYNU (kynurenine), LRRK2 (The leucine-rich repeat kinase 2), TCL1A (The T cell leukemia-lymphoma 1 TCL1), TJP2 (tight junction protein 2), BCLAF1 (Bcl-2-associated transcription factor 1), RHBDD1 (The rhomboid domain containing 1), MEF2C (the myocyte enhancer factor-2C), IDO1 (indoleamine 2,3-dioxygenase 1), NFkB1, POGZ (pogo transposable element-derived protein with zinc finger domain), and inhibitor of nuclear factor kappa B kinase subunit B IKBKB (Fig. 4D). Upregulation of NF-κB gene expression was confirmed by qPCR, as shown in Fig. 4E, NF-κB mRNA was 3-folds higher in HIV-1 infected CD4^+^ T cells when cocultured with CECs relative to HIV-1 infected CD4^+^ T cells alone.

10.1128/mBio.02767-19.3 FIG S3 (A) Selected highly upregulated and downregulated genes in HIV-infected CD4^+^ T cells in the presence of CECs versus HIV-infected CD4^+^ T cells alone. (B) Gene Ontology analysis of the biological process of the transcriptome profile of cocultured CD4^+^ T cells with CECs. (C) Cumulative data showing mRNA expression levels for arginase-2 (Arg-2) in the cord blood CECs from healthy and non-IBD donors versus ulcerative colitis or Crohn’s disease patients. (D) Cumulative data showing mRNA expression levels for arginase-2 (Arg-2) in the placenta CECs from healthy and non-IBD donors versus patients with ulcerative colitis or Crohn’s disease. Download FIG S3, JPG file, 0.1 MB. Copyright © 2019 Namdar et al. 2019 Namdar et al. This content is distributed under the terms of the Creative Commons Attribution 4.0 International license.

### CECs enhance HIV infection via ROS.

**FIG 5:**
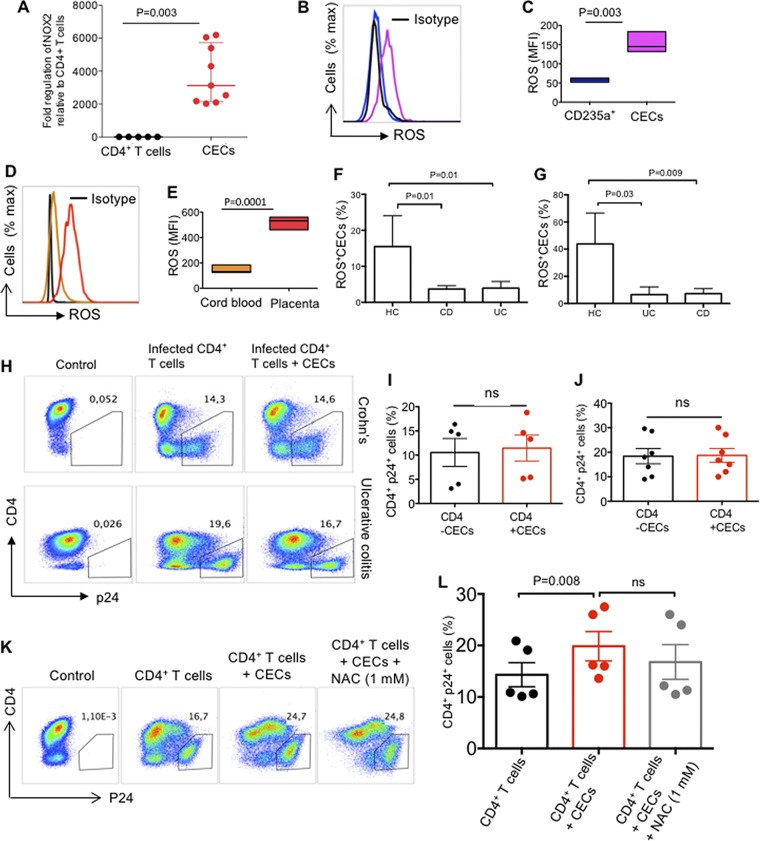
CECs enhance HIV infection through ROS. (A) NOX2 gene expression in CECs versus CD4^+^ T cells. (B) Representative histogram showing ROS expression (RBCs [blue line] or CECs [purple line]) or the isotype control (black line). (C) Mean fluorescent intensity (MFI) of ROS among RBCs (CD235a^+^) and CECs. (D) Representative histogram showing ROS expression in CECs from cord blood (orange line) or placenta (red line) and the isotype control (black line). (E) MFI of ROS among the cord blood or placenta CECs. (F) Cumulative data showing the percentage of ROS^+^ CECs in the cord blood or (G) the placenta from healthy controls (HC) versus patients with Crohn’s disease (CD) or ulcerative colitis (UC). (H) Representative flow cytometry plots showing HIV infection in activated CD4^+^ T cells in the presence or absence of cord blood CECs. (I and J) Cumulative data showing the percentage of p24 in activated CD4^+^ T cells in the presence or absence of CECs from the cord blood of newborns to CD (I) or UC (J) mothers. ns, not significant. (K) Representative flow cytometry plots showing HIV infection in the cord blood CD4^+^ T cells in the presence or absence of CECs and *N*-acetyl cysteine (NAC). (L) Cumulative data showing the percentage of p24 in activated CD4^+^ T cells in the presence/absence of CECs and/or NAC.

**FIG 6:**
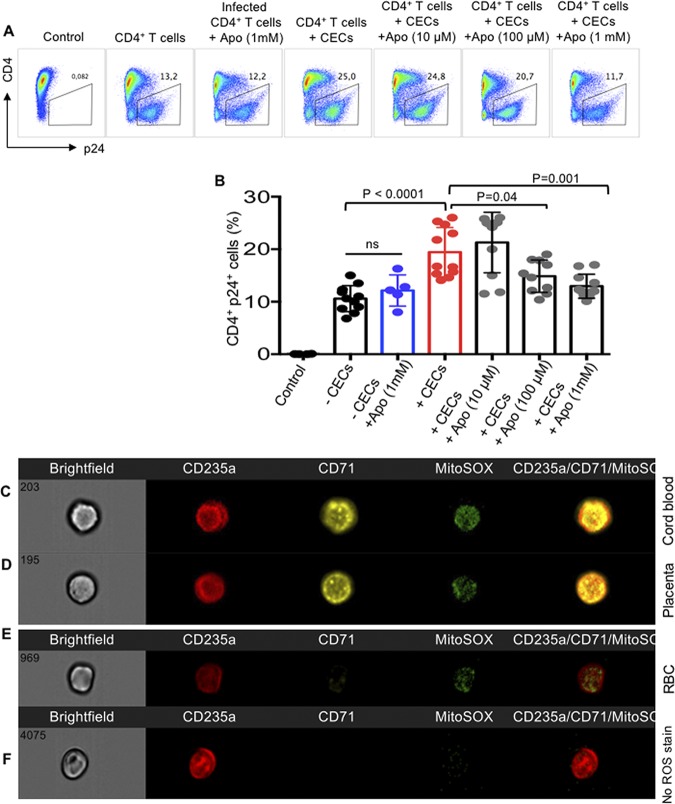
Apocynin abrogates CEC-mediated HIV infection by ROS. (A) Representative flow cytometry plots showing HIV infection in the cord blood CD4^+^ T cells in the presence or absence of CECs and apocynin (Apo). (B) Cumulative data showing the percentage of p24 in activated CD4^+^ T cells in the presence/absence of CECs and/or Apo. (C to F) Representative images collected using an Amnis ImageStream Mark II showing MitoSOX in the cord blood (C) or placenta (D) CECs and RBCs (E) compared to the ROS isotype control (F).

It has been reported that RBCs secrete endogenous ROS (30) and the hydrogen peroxide produced by these cells exhibits various immunological functions (31). More importantly, Gene Ontology analysis of the biological process of the transcriptome profile of cocultured CD4^+^ T cells with CECs revealed upregulation of cellular response to oxygen-containing compounds and NF-κB signaling (Fig. S3B). Therefore, we performed qPCR on CECs for the expression of NOX family genes which comprises of seven paralogous, including NOX1-5 and dual oxidase (DUOX1/2) (32). Interestingly, we observed a prominent gene expression of NOX2 in CECs (Fig. 5A), while NOX paralogues (NOX 1, 3, 4, 5, DUOX1, and 2) were undetectable. We further compared ROS production by RBCs (CD235a) versus CECs either from the cord blood or placenta. We found that CECs compared to their mature counterparts produced significantly higher levels of ROS (Fig. 5B and C), and CECs from the placenta express significantly higher levels of ROS compared to their counterparts in the cord blood (Fig. 5D and E). In agreement with our previous report, we observed lower ROS expression by CECs from the cord blood and placenta of inflammatory bowel disease (IBD) patients compared to healthy controls (12) (Fig. 5F and G). This indicates a dichotomy in ROS production by CECs from IBD patients compared to the healthy controls. However, there was no significant difference in mRNA expression levels for arginase-2 in CECs isolated from the cord blood or placenta of IBD versus healthy donor samples (Fig. S3C and D). We further aimed to determine the role of NADPH oxidases (NOX) complex, which is involved in ROS generation. Due to the dichotomy in ROS production by CECs from IBD patients compared to healthy donors, we decided to perform HIV-1 infection assays using autologous CD4^+^ T cells and CECs from the cord blood of IBD patients. Interestingly, we found lack of enhanced HIV replication in autologous CD4^+^ T cells by CECs obtained from the cord blood of IBD donors (Fig. 5H to J). Next, we assessed whether downregulation of ROS activity by ROS inhibitors could mitigate HIV infection in CD4^+^ T cells. To evaluate this, HIV-infected CD4^+^ T cells in the presence of CECs were exposed to two types of ROS inhibitors: *N*-acetyl cysteine (NAC) and apocynin (Apo). The universal ROS blocker (NAC) failed to inhibit the enhanced viral replication by CECs in CD4^+^ T cells even at a 1 mM concentration (Fig. 5K and L; see Fig. S4A in the supplemental material). In contrast, Apo, which is the NADPH-dependent ROS inhibitor, abrogated the enhanced HIV-1 infection/replication in CD4^+^ T cells by CECs (Fig. 6A and B). This observation suggested that CECs might release mitochondrial ROS, which was confirmed by the mitochondrial superoxide (MitoSOX) indicator (Fig. 6C to F). In addition, we confirmed that CECs from either the cord blood (Fig. 6C) or the placenta (Fig. 6D) had higher mitochondrial superoxide compared to RBCs (Fig. 5E). Lower ROS content in RBCs could explain the lack of enhanced HIV-1 replication by these cells (Fig. 2J and K), which supports the important role of ROS in this process. This was further confirmed as Apo significantly reduced MitoSOX expression in CECs, but this was not the case for NAC (Fig. S4B and C). Although surprising, our findings indicate that the mitochondrial ROS released by CECs can be scavenged by Apo but not by NAC.

10.1128/mBio.02767-19.4 FIG S4 (A) Cumulative data showing the percentages of HIV-infected CD4^+^ T cells in the absence/presence of CECs and different concentrations of NAC after 4 days measured by flow cytometry. (B) Representative ImageStream plots showing MitoSOX expression levels in CECs in the presence of Apo (1 mM) or NAC (1 mM). (C) Cumulative data presenting MitoSOX expression levels in CECs without an ROS scavenger or with either Apo or NAC. Download FIG S4, JPG file, 0.08 MB. Copyright © 2019 Namdar et al. 2019 Namdar et al. This content is distributed under the terms of the Creative Commons Attribution 4.0 International license.

### CECs express significantly greater levels of DARC and CD35 compared to RBCs.

**FIG 7:**
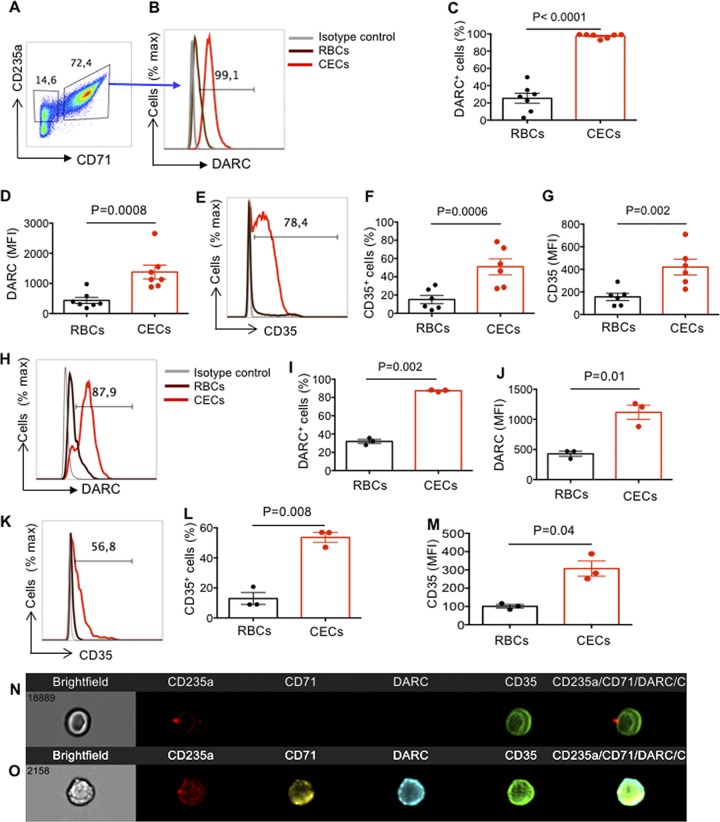
DARC and CD35 are highly expressed on CECs. (A) Representative flow cytometry plots showing the gating strategy for CECs (CD71^+^ CD235a^+^) versus RBCs (CD235a^+^). (B) Representative histogram showing DARC expression on CECs from the cord blood (red line) or RBCs (brown line) and the isotype control (black line). (C and D) Cumulative data showing the percentage of DARC expression (C) or the MFI of DARC expression (D) on RBCs versus CECs from the cord blood. (E) Representative histogram showing CD35 expression on CECs from the cord blood (red line) or RBCs (brown line) and the isotype control (black line). (F and G) Cumulative data showing the percentage of CD35 expression (F) or the MFI of CD35 expression (G) on RBCs versus CECs from the cord blood. (H) Representative histogram showing DARC expression on CECs from the placenta (red line) or RBCs (brown line) and isotype control (black line). (I and J) Cumulative data showing the percentage of DARC expression (I) or the MFI of DARC expression (J) on RBCs versus CECs from the placenta. (K) Representative histogram showing CD35 expression on CECs from the placenta (red line) or RBCs (brown line) and isotype control (black line). (L and M) Cumulative data showing the percentage of CD35 expression (L) or the MFI of CD35 expression (M) on RBCs versus CECs from the placenta. (N and O) Representative images collected using an Amnis ImageStream Mark II showing DARC and CD35 expression on RBCs (N) or CECs (O) from the cord blood.

The role of DARC on RBCs in mediating HIV-1 *trans*-infection of the target cells has already been documented (18). Therefore, we decided to determine whether CECs similar to their older siblings express DARC. Interestingly, we observed nearly 100% of CECs express DARC compared to approximately 20% of RBCs (Fig. 7A to C). Additionally, the intensity of DARC was significantly higher on CECs compared to RBCs (Fig. 7D). Another molecule of interest was CD35 (CR-1), the opsonized role of which for HIV particles via HIV/anti-HIV immune complexes and complement factor C3b binding has already been demonstrated (33). Therefore, we measured the expression level of CR-1 on both RBCs and CECs. Interestingly, we found substantial surface expression of CR-1 on CECs, which was significantly higher in terms of percentage and intensity compared to RBCs (Fig. 7E to G). A similar pattern of expression was observed for both DARC (Fig. 7H to J) and CD35 (Fig. 7K to M) on placental CECs. Finally, ImageStream analysis confirmed the higher expression of DARC and CD35 on CECs versus RBCs (Fig. 7N and O). Thus, our observations indicate prominent expression of DARC and CD35 on CECs.

### CECs are more efficient than RBCs at mediating HIV-1 *trans*-infection of CD4^+^ T cells.

**FIG 8:**
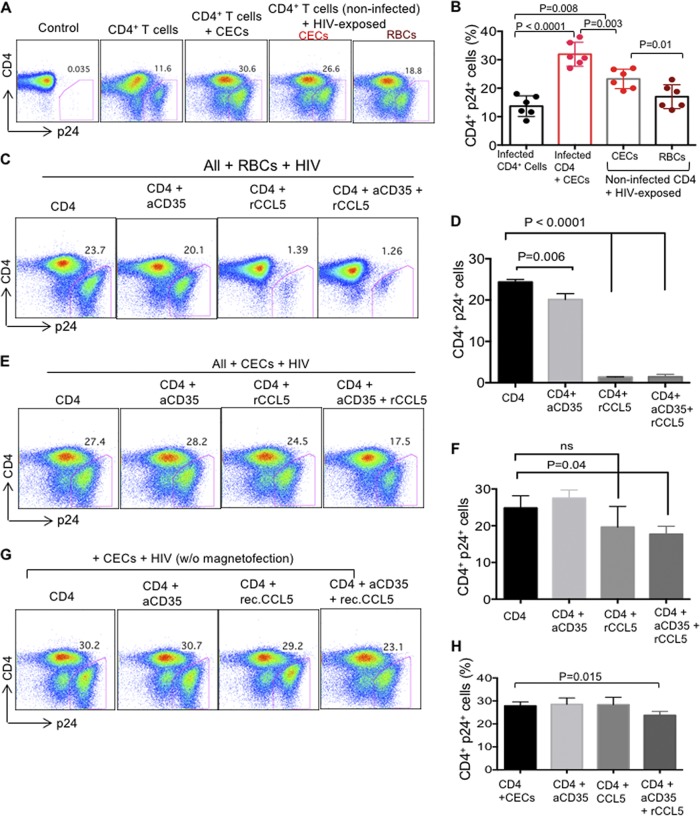
CECs mediate *trans*-infection of HIV-1 to CD4^+^ T cells. (A) Representative flow cytometry plots showing CD4^+^ T cells from the cord blood following infection with the R5-tropic isolate in the absence/presence of CECs and also CECs or RBCs exposed to HIV and then cocultured with noninfected but activated CD4^+^ T cells. (B) Cumulative data showing the percentage of p24 in CD4^+^ T cells in the absence/presence of CECs and also CECs/RBCs exposed to HIV then cocultured with noninfected but activated CD4^+^ T cells. (C) Representative flow cytometry plots showing HIV-1 (R5-tropic) *trans*-infection by RBCs of activated CD4^+^ T cells in the presence of anti-CD35 antibody (10 μg/ml), rCCL-5 (100 nM), or their combination (anti-CD35 [10 μg/ml] and rCCL-5 [100 nM]). (D) Cumulative data showing the percentage of CD4^+^ p24^+^ T cells following HIV (R5-tropic) *trans*-infection by RBCs of activated CD4^+^ T cells in the presence of anti-CD35 antibody (10 μg/ml), rCCL-5 (100 nM), or their combination (anti-CD35 [10 μg/ml] and rCCL-5 [100 nM]). (E) Representative flow cytometry plots showing HIV-1 (R5-tropic) *trans*-infection by CECs of activated CD4^+^ T cells in the presence of anti-CD35 antibody (10 μg/ml), rCCL-5 (100 nM), or their combination (anti-CD35 [10 μg/ml] and rCCL-5 [100 nM]). (F) Cumulative data showing the percentage of CD4^+^ p24^+^ T cells following HIV (R5-tropic) *trans*-infection by CECs of activated CD4^+^ T cells in the presence of anti-CD35 antibody (10 μg/ml), rCCL-5 (100 nM), or their combination (anti-CD35 [10 μg/ml] and rCCL-5 [100 nM]). (G) Representative flow cytometry plots showing infected CD4^+^ T cells in the presence of CECs alone or in the presence of CECs plus anti-CD35 antibody (10 μg/ml), CCL-5 (100 nM), or their combination (anti-CD35 [10 μg/ml] and rCCL-5 [100 nM]) without magnetofection. (H) Cumulative data showing the percentage of CD4^+^ p24^+^ T cells in the presence of CECs alone or in the presence of CECs plus anti-CD35 antibody (10 μg/ml), rCCL-5 (100 nM), or their combination (anti-CD35 [10 μg/ml] and rCCL-5 [100 nM]) without magnetofection. A minimum of four to five experiments/samples were used for the data presented in panels D, F, and H.

To determine whether CECs mediate HIV-1 *trans*-infection of CD4^+^ T cells as reported for RBCs (18, 34), we incubated CECs or RBCs with HIV-1 (exactly similar to the methods used for infecting CD4^+^ T cells). The cells were then extensively washed and cocultured with autologous uninfected CD4^+^ T cells. We found that preexposure of CECs or RBCs to HIV-1 (R5-tropic) resulted in carriage of the virus and *trans*-infection of CD4^+^ T cells when analyzed 4 days post-coculture (Fig. 8A and B). The infection rate in *trans*-infected CD4^+^ T cells was slightly lower to the infection rate of CD4^+^ cells when cocultured with CECs but significantly higher than infected CD4^+^ T cells in the absence of CECs (Fig. 8A and B). Interestingly, transmission of HIV-1 by CECs was more efficient compared to that in their older siblings (Fig. 8A and B). Furthermore, we decided to evaluate the effects of DARC and CD35 blockade on HIV *trans*-infection by CECs since such a role for RBCs has been reported (18, 33). First of all, we showed that the addition of anti-CD35 antibody and/or rCCL-5 (to compete for DARC) had no significant effects on HIV infection/replication in CD4^+^ T cells alone (see Fig. S5A and B in the supplemental material). Then, we decided to determine if we can interfere with the interaction of HIV with its potential targets (DARC and CD35) on RBCs as previously described elsewhere (18). First, RBCs were preincubated with either anti-CD35 (10 μg/ml) or rCCL-5 (1 or 100 nM) for 2 h prior to the viral exposure. Then RBCs were subjected to magnetofection using an R5-tropic HIV-1 isolate. Although preexposure to anti-CD35 antibody moderately reduced HIV *trans*-infection from RBCs of CD4^+^ T cells, rCCL-5 at 100 nM almost abolished the ability of RBCs to *trans*-infect CD4^+^ T cells with HIV-1 (Fig. 8C and D). However, no synergistic effect was observed for the combination of anti-CD35 antibody and rCCL-5: rCCL-5 at a lower concentration (1 nM) despite the previous report (18) did not inhibit HIV *trans*-infection from RBCs of CD4^+^ T cells (data not shown). In parallel, similar experiments were performed on CECs, which revealed interesting results. First, we found that the anti-CD35 antibody (10 μg/ml) alone was unable to block HIV *trans*-infection from CECs of CD4^+^ T cells (Fig. 8E and F). Second, we observed that although rCCL-5 (100 nM) prevented HIV *trans*-infection from RBCs by ∼95%, this was not the case for CECs (Fig. 8E and F). However, the combination of rCCL-5 and anti-CD35 antibody moderately reduced HIV *trans*-infection both with magnetofection (Fig. 8E and F) and without magnetofection (Fig. 8G and H). To exclude possible interference of the serum in culture medium on the activity of the anti-CD35 antibody, we performed the assay with CD35 blocked in the absence of serum. However, we did not observe any reduction in the HIV-1 *trans*-infection of CD4^+^ T cells (Fig. S5C and D). Finally, we found that preexposure of CECs to HIV-1 also *trans*-infected nonactivated CD4^+^ T cells, although at a lower rate, as predicted (see Fig. S6A in the supplemental material). Our data indicate that CECs were efficiently able to *trans*-infect uninfected CD4^+^ T cells with HIV-1, even in the presence of rCCL-5 and/or the anti-CD35 antibody. However, anti-CD35 antibody to some extent but rCCL-5 almost abrogated HIV-1 *trans*-infection from RBCs of uninfected CD4^+^ T cells. These observations suggest a differential mechanism of HIV-1 *trans*-infection of target cells by CECs versus RBCs.

10.1128/mBio.02767-19.5 FIG S5 (A) Representative flow cytometry plots and (B) Cumulative data showing the percentage of CD4^+^ p24^+^ T cells in the presence of CECs alone or in the presence of CECs plus anti-CD35 antibody (10 μg/ml), rCCL-5 (100 nM), or their combination (anti-CD35 [10 μg/ml] and rCCL-5 [100 nM]) using magnetofection. (C) Flow cytometry plots showing the HIV infection rate in CD4^+^ T cells in the presence/absence of CECs or following exposure of CECs to HIV in the presence of anti-CD35 (10 μg/ml) using serum-free culture medium. (D) Cumulative data showing the HIV infection rate in CD4^+^ T cells in the presence/absence of CECs or following exposure of CECs to HIV in the presence of anti-CD35 (10 μg/ml) using serum-free culture medium. Download FIG S5, JPG file, 0.08 MB. Copyright © 2019 Namdar et al. 2019 Namdar et al. This content is distributed under the terms of the Creative Commons Attribution 4.0 International license.

10.1128/mBio.02767-19.6 FIG S6 (A) Representative flow cytometry plots showing HIV infection in nonactivated CD4^+^ T cells following coculture with HIV-exposed CECs. (B and C) Representative plots (B) and cumulative data (C) showing HIV *trans*-infection from RBCs of CD4^+^ T cells in the absence or presence of anti-CD235a antibody. (D) Representative flow plots showing the purity of CD4^+^ T cells pre- and postisolation. (E) Representative plots showing the purity of CECs postisolation. (F) Flow cytometry plots showing the percentage of viability of CD4^+^ T cells in the absence or presence of CECs. Download FIG S6, JPG file, 0.08 MB. Copyright © 2019 Namdar et al. 2019 Namdar et al. This content is distributed under the terms of the Creative Commons Attribution 4.0 International license.

### HIV-1 preferentially binds to CD235a on CECs, which facilities HIV *trans*-infection.

**FIG 9:**
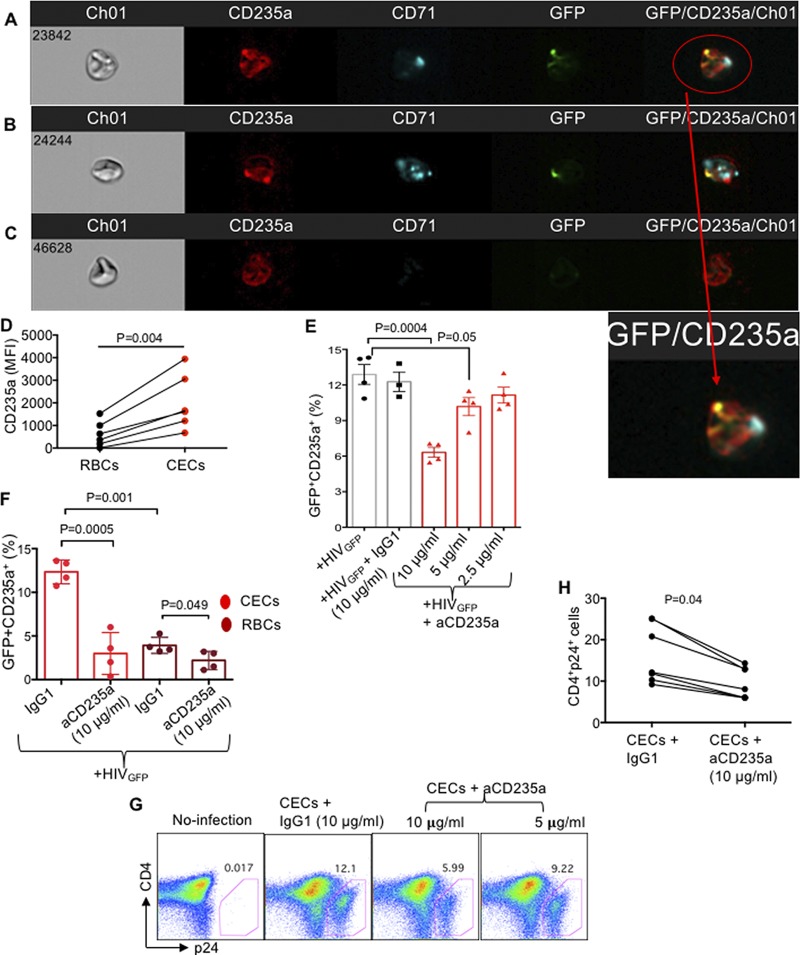
HIV-1 binds preferentially to CD235a on CECs but weakly on RBCs. (A to C) Representative ImageStream plots showing interaction of CECs (A and B) with GFP-marked HIV versus RBCs (C). (D) Cumulative data showing MFI of CD235a on CECs versus RBCs. (E) Cumulative data showing the percentage of GFP^+^ CD235a on CECs in the presence of IgG1 isotype control antibody (10 μg/ml) or different concentrations of anti-CD235a antibody. (F) Cumulative data representing the percentage of GFP^+^ CD235a on CECs or RBCs in the presence of the IgG1 (10 μg/ml) isotype or anti-CD235a antibody. (G) Representative flow cytometry plots showing *trans*-infection of CD4^+^ T cells by CECs after exposure to HIV (X4-tropic) in the presence or absence of anti-CD235a antibody. (H) Cumulative data showing *trans*-infection of CD4^+^ T cells by CECs after exposure to HIV (X4-tropic) in the presence or absence of anti-CD235a antibody.

Since blockade of CD35 and addition of rCCL-5 did not abrogate CECs-mediated HIV *trans*-infection of CD4^+^ T cells, we decided to investigate the interaction of HIV with CECs using an R5-tropic green fluorescent protein (GFP)-marked virus. Using ImageStream, we observed that HIV interacts more strongly with CECs (Fig. 9A and B) compared to RBCs (Fig. 9C). Most interestingly, we found colocalization of CD235a with GFP on CECs, which suggests the interaction of HIV with CD235a (Fig. 9A and B). However, this was not prominent for RBCs (Fig. 9C). Significantly higher MFI for CD235a on CECs versus RBCs was suggested as a possible mechanism for the enhanced interaction of CECs with CD235a (Fig. 9D). These observations were further supported by using the anti-CD235a blocking antibody (Fig. 9E). Although anti-CD235a antibody significantly reduced the binding of GFP^+^ virus to both CECs and RBCs, the interaction of GFP^+^ virus with CECs was significantly higher (>2-fold) than its interaction with RBCs (Fig. 9F). Additional studies were performed to determine whether the anti-CD235a antibody can interfere with HIV-1 (R5-tropic) *trans*-infection of CD4^+^ T cells by CECs or RBCs. Interestingly, we found that the anti-CD235a antibody significantly abrogated the *trans*-infection of HIV-1 by CECs to already activated autologous CD4^+^ T cells (Fig. 9G and H). Nevertheless, this antibody at the same concentration did not prevent the *trans*-infection of HIV-1 by RBCs (Fig. S6B and C). Although the results in Fig. 9F suggested a slight interaction of GFP-marked virus with CD235a on RBCs, the anti-CD235a antibody did not impact HIV-1 transmission from RBCs to CD4^+^ T cells (Fig. S6A and B). This discrepancy might be related to the incubation time: overnight for the GFP^+^ virus versus 4 days of culture for the p24 study. Thus, our data suggest CD235a as a potential binding receptor for HIV-1 and provide a novel insight into the capabilities of CECs versus RBCs in HIV-1 *trans*-infection of target cells.

### CECs but not RBCs harbor HIV-1 and *trans*-infect uninfected CD4^+^ T cells.

**FIG 10:**
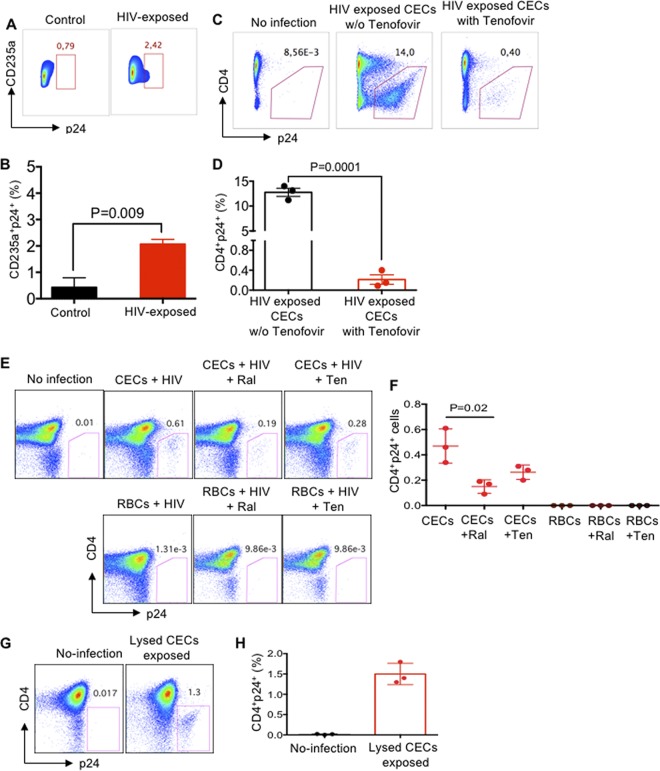
CECs harbor infective HIV virions. (A) Representative flow cytometry plots showing p24 in HIV-exposed CECs versus the control. (B) Cumulative data showing the percentage of p24 in HIV-exposed CECs versus controls. (C) Representative flow cytometry plots showing HIV *trans*-infection of CD4^+^ T cells following treatment of HIV (X4-tropic)-exposed CECs to tenofovir (4.3 μg/ml) for 16 h. (D) Cumulative data showing HIV *trans*-infection by CECs of CD4^+^ T cells following treatment of HIV (X4-tropic)-exposed CECs to tenofovir for 16 h. (E) Representative plots showing HIV *trans*-infection of CD4^+^ T cells following treatment of HIV (X4-tropic)-exposed CECs or RBCs with either tenofovir (Ten [4.3 μg/ml]) or raltegravir (Ral [10 μg/ml]) for 74 h. (F) Cumulative data on HIV *trans*-infection of CD4^+^ T cells following treatment of HIV (X4-tropic)-exposed CECs or RBCs to either tenofovir (4.3 μg/ml) or raltegravir (10 μg/ml) for 74 h. (G and H) Representative plots (G) and cumulative data (H) showing HIV-1 infection of CD4^+^ T cells following exposure to lysed CECs.

We exposed CECs to the X4-tropic HIV-1 isolate according to our routine infection protocol (25). Three days later, the infection rate in CECs was quantified by measuring p24 (Fig. 10A and B). Although the majority of the cord blood CECs lack nuclei, about 5% of them still have nuclei, which may explain that HIV can reside and possibly replicate in CECs (Fig. 10A and B).

To further understand the physiological relevance of CEC-mediated HIV-1 *trans*-infection in HIV patients on ART, CECs were exposed to HIV for 40 min and then washed extensively and incubated with a physiologically relevant dose of tenofovir (4.3 μg/ml) for 16 h. CECs were then washed and cocultured with uninfected but activated autologous CD4^+^ T cells for 3 days. Our observations indicated that the virus associated with CECs even in the presence of tenofovir was capable of *trans*-infecting HIV target cells (Fig. 10C and D), which suggests HIV may reside in CECs. Further studies were designed to determine if life-cycle-blocking ARTs block HIV *trans*-infection by CECs. For these studies, RBCs and CECs were exposed to HIV-1 (R4-tropic) for 40 min using magnetofection. Then, cells were extensively washed and incubated with tenofovir (4.3 μg/ml) and raltegravir (10 μg/ml) or without drugs for 74 h, after which cells were washed and added to already activated autologous CD4^+^ T cells. Interestingly, we observed that CECs but not RBCs, even in the presence of tenofovir and raltegravir, remained infectious and *trans*-infected CD4^+^ T cells (Fig. 10E and F). To reconfirm this *in vivo*, CECs were obtained from HIV-1-infected but ART-naive individuals. Isolated CECs were extensively washed and lysed using RBC lysis buffer (2 min). The supernatant was added to autologous CD4^+^ T cells for 40 min and analyzed 3 days later. Interestingly, we found *trans*-infection of CD4^+^ T cells by lysed CECs (Fig. 10G and H), indicating that CECs may serve as HIV carriers or reservoirs in HIV-infected individuals.

### Characterization of CECs obtained from the peripheral blood of patients.

**FIG 11:**
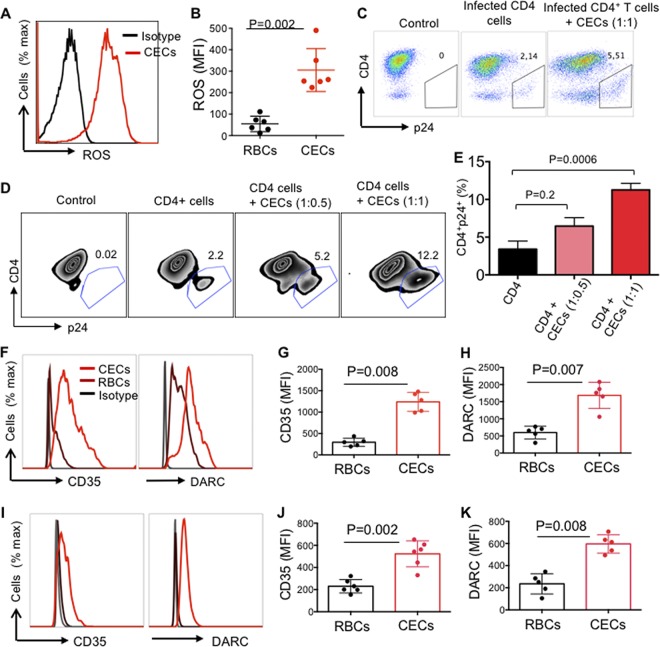
CECs from the peripheral blood of HIV patients and anemic individuals enhance HIV infection in autologous CD4^+^ T cells. (A) Representative histogram showing ROS expression in CECs of an HIV-infected patient (red line) and isotype control (black line). (B) Cumulative data showing comparison of ROS by CECs versus RBCs in HIV-infected individuals. (C) Representative flow cytometry plots showing HIV infection in activated CD4^+^ T cells alone or in the presence of autologous CECs obtained from an HIV-infected individual. (D) Representative flow cytometry plots showing HIV infection in activated CD4^+^ T cells alone or in the presence of autologous CECs obtained from an anemic individual. (E) Cumulative data showing the percentage of CD4^+^ p24^+^ T cells in the absence or presence of CECs from anemic individuals in a dose-dependent manner. (F) Representative histogram showing CD35 and DARC expression in CECs (red line), RBCs (brown line), and isotype control (black line) from an HIV-infected individual. (G and H) Cumulative data presenting the MFI for CD35 (G) and DARC (H) in RBCs versus CECs in HIV-infected individuals. (I) Representative histogram showing CD35 and DARC expression in CECs (red line), RBCs (brown line), and isotype control (black line) from an anemic individual. (J and K) Cumulative data presenting the MFI for CD35 (J) and DARC (K) in RBCs versus CECs in anemic individuals.

As shown in Fig. 1, we found CECs become abundant in the blood of HIV-infected individuals versus healthy controls. Then we decided to further study their similarities and differences with cord blood CECs. We found that CECs from the peripheral blood of HIV patients generate ROS (Fig. 11A and B). Since expanded CECs in HIV-infected individuals produce ROS, we reasoned these cells similar to the cord blood CECs might enhance HIV-1 infection in CD4^+^ T cells. In agreement, we found enhanced HIV-1 infection in CD4^+^ T cells when cocultured with autologous CECs *in vitro* (Fig. 11C). We also found that CECs isolated from the blood of anemic individuals enhance HIV-1 infection/replication when cocultured with autologous CD4^+^ T cells (Fig. 11D and E). Finally, we found that CECs obtained from HIV^+^ (Fig. 10F to H) and anemic (Fig. 10I to K) individuals express substantial levels of DARC and CD35 compared to RBCs.

## DISCUSSION

The association of HIV-1 with cell surface markers on erythrocytes has not been free of controversy. Erythrocytes from HIV-1-infected individuals were reported to contain the cell-bound virus and proposed to represent a novel HIV-1 reservoir (35). However, this concept was later disputed and the reservoir hypothesis for erythrocytes was challenged (36). Subsequent studies confirmed that CR1 (CD35) and DARC were responsible for HIV-1 binding to the erythrocytes and virus *trans*-infection of target cells (18, 33). Despite these reports, the interaction of erythroid precursors with HIV-1 has never been investigated and has remained a mystery. We have recently reported an abundance of erythroid precursors (CECs) in the cord blood, placental tissues, and peripheral blood of pregnant mothers (12, 14, 17). We further indicated that CECs possess a wide range of immunological properties (37). Here, for the first time, we report the expansion of CECs in the peripheral blood of HIV-infected individuals. More importantly, we found a positive correlation between the frequency of CECs and the plasma viral load in ART-naive patients, which suggests an important role for CECs in HIV pathogenesis. We used umbilical cord blood, which contains higher percentage of CECs than blood of HIV patients, to investigate their role in HIV infection/replication. Our work reveals a novel role for CEC-mediated enhanced HIV-1 infection/replication in CD4^+^ T cells. We demonstrate that CECs from human cord blood or placental tissues in a dose-dependent manner exacerbate HIV-1 replication and infection, by both R5- and X4-tropic viral strains, in autologous CD4^+^ T cells. Although this process was most marked for already activated CD4^+^ T cells, CECs significantly enhanced HIV-1 infection in nonactivated CD4^+^ T cells as well. Furthermore, we discovered that CECs do not require cell-cell interactions but via soluble factors enhance HIV-1 infection/replication in autologous CD4^+^ T cells. To understand the mechanism associated with these observations, we found that the production of arginase-2 and TGF-β (27) by CECs did not enhance HIV-infection. Therefore, to determine the mechanism(s) associated with the enhanced HIV infection/replication, RNA-seq analysis was performed. We found the transcriptome profile of infected CD4^+^ T cells cocultured with CECs was separate from that of the infected CD4^+^ T cells in the absence of CECs. Gene Ontology analysis of the biological process for the transcriptome profile revealed the upregulation of cellular response to oxygen-containing compounds and upregulation of NF-κB signaling in CD4^+^ cells when cocultured with CECs. Our subsequent studies revealed that CECs have substantial levels of NOX2 mRNA, while other NOX paralogues (NOX1, -3, -4, and -5 and DUOX1 and -2) were undetectable. Although endogenous ROS generation by RBCs has been documented (38), we observed that CECs have significantly higher ROS production capacity compared to their mature counterparts. More importantly, we observed that CECs release mitochondrial ROS, the function of which can be abrogated by the ROS scavenger apocynin but not by *N*-acetyl cysteine. Interestingly, we observed that CECs, when obtained from the cord blood of mothers with IBD, did not enhance HIV-1 infection in autologous CD4^+^ T cells—possibly because of their impaired ROS production ability (12). Other factors and cofounders associated with IBD samples may impact the functionality of CECs, which requires further investigations. Subsequently, we found elevated NF-κB gene expression in CD4^+^ T cells when cocultured with CECs, suggesting enhanced NF-κB expression by ROS. A difficulty in establishing ROS signaling is that ROS often functions differently in a given pathway (e.g., upstream or downstream). This seems to be the case with regard to the role of ROS in the NF-κB pathway. For example, ROS stimulates the NF-κB pathway in the cytoplasm but not in the nucleus (39). In this report, we believe that ROS released by CECs may activate NF-κB (40) and ROS production by CECs might be an essential step leading to NF-κB activation. In agreement, the IKBKB gene was highly upregulated (>8-fold) in HIV-1-infected CD4^+^ T cells when exposed to CECs compared to in HIV-1-infected CD4^+^ T cells alone.

Activation of NF-κB is considered a crucial step in HIV-1 replication (41, 42). Therefore, we believe increased intracellular levels of NF-κB may permit high levels of HIV-1 gene expression and could thus provide a favorable environment for HIV replication in CD4^+^ T cells in the presence of ROS. In addition to NF-κB upregulation, a wide range of other genes were highly upregulated in CD4^+^ T cells when cocultured with CECs. Among them, the tTg gene was the most upregulated gene, which may be a natural and nonspecific mechanism of CD4^+^ T cell defense (43), aimed at reducing viral spread.

Another important upregulated gene was the AQP9 gene, expression of which in peripheral blood mononuclear cells (PBMCs) of HIV-infected individuals has already been reported (44). Although its role in HIV infection is unknown, we suggest AQP9 may act as an ROS scavenger to prevent T cells apoptosis (29). Similarly, upregulation of the MYOF gene in HIV-1-infected cells has been documented (45). We propose that MYOF participates in the repair of the plasma membrane following the budding of a multitude of HIV-1 virions, allowing the cell to live longer and to produce more viruses, which is the case in our study. The ASAP1 gene was another gene highly upregulated in HIV-1-infected CD4^+^ T cells when exposed to CECs. Although its role in HIV-1 infection is unknown, overexpression of ASAP1 enhances cancer cell proliferation and invasion (46). There is a possibility that the upregulation of ASAP-1 and TCL1 inhibits apoptosis (47) of HIV-1-infected CD4^+^ T cells, enabling them to produce more viral particles. Besides, we observed upregulation of the Lck gene in CD4^+^ T cells exposed to CECs, which suggests Lck may facilitate HIV-1 assembly and release from the T cell plasma membrane as reduction of Lck has been reported to reduce HIV replication in CD4^+^ T cells (48). The BCLAF1 gene was another gene highly upregulated in infected CD4^+^ T cells when cocultured with CECs, which may act as an HIV-1-restricting factor as reported for human cytomegalovirus (49).

The role of the LRRK2 gene in HIV-1 pathogenesis is unknown; however, it plays an important role against intracellular pathogens such as Mycobacterium leprae (50), Salmonella enterica serovar Typhimurium (51), and Listeria monocytogenes (52) via inflammasome activation (53). Thus, we suggest it may play a similar role against HIV-1 infection. Notably, IDO1 and KYNU genes were profoundly upregulated in CD4^+^ T cells when CECs were present. IDO-1-driven generation of kynurenine has an immune regulatory function, and IDO-1 appears to prevent viral protein production (54). In this context, we believe IDO-1 and KYNU may act to limit viral replication, which is indicative of the high viral production in CD4^+^ T cells cocultured with CECs. Another important gene to mention was the PogZ gene. Interaction of PogZ with HIV achieves efficient integration, which results in increased infectivity (55). Collectively, our data suggest that CECs via ROS production upregulate a wide range of genes, and some of them may explain enhanced viral infection/replication in CD4^+^ T cells.

Another aspect of our study was to determine whether CECs via binding to HIV-1 can *trans*-infect uninfected CD4^+^ T cells with the virus. Although the exact mechanism of HIV-1 binding to RBCs is not fully understood, it is reported that HIV-1 binds to CR1 (CD35) (33) and DARC on RBCs (18, 34). Binding of HIV to CR1/DARC demonstrates that HIV might exploit CR1/DARC to its advantage as a unique niche that enables viral survival and *trans*-infection of HIV target cells. It was striking that both DARC and CR1 were highly expressed on CECs compared to their mature counterparts in terms of intensity and frequency. Thus, we speculated that these two receptors might come into play together to confer an accelerated rate of HIV *trans*-infection of CD4^+^ T cells. We observed that preincubation of CECs with HIV-1 enables the virus to bind to these cells and subsequently CECs *trans*-infect CD4^+^ T cells with HIV-1. To establish that these effects were mediated specifically by DARC and/or CR1, we investigated whether rCCL-5 a chemokine ligand for DARC, or antibody against CR1 can compete for the adherence of HIV to DARC/CR1 on CECs. In agreement with the previous reports that RBC-bound HIV occurs predominantly via DARC (18, 34), we found that rCCL-5 substantially prevented HIV (R5-tropic) *trans*-infection of CD4^+^ T cells. However, this effect was not observed at the reported concentration of 1 nM but at a 100× higher concentration. Interestingly, rCCL-5 failed to prevent adherence of HIV to CECs. Although CECs have the greater intensity for DARC on their surface, and almost 100% of them express DARC compared to approximately 20% of RBCs, we suggest DARC might not be the dominant receptor for HIV. Our results thus appear to be compatible with previous reports that have challenged the binding of HIV to DARC as a crucial factor in HIV pathogenesis, when a lower concentration of rCCL-5 (1 nM) was used (56–58). Similarly, despite previous reports for the role of CR1 in RBCs for enhanced HIV infectivity of target cells (33), in our hands, anti-CR1 antibody had moderate preventive effects on HIV *trans*-infection by RBCs. It also failed to block *trans*-infection by HIV of CD4^+^ T cells by CECs. Our observations do not exclude DARC and CR1 as possible RBC binding sites since a partial reduction in HIV *trans*-infection occurred when the anti-CR1 antibody was applied. More importantly, rCCL-5 almost abrogated the HIV *trans*-infection by RBCs. However, the highly efficient transfer of HIV from CECs to CD4^+^ T cells even in the presence of rCCL-5 and CR1 blockade suggests another potential HIV binding molecule on CECs. Our further studies revealed that HIV interacts with CD235a, as reported for the hepatitis A and influenza viruses (59, 60). More importantly, we observed more efficient interaction of HIV with CD235a on CECs compared to RBCs. This might be explained by a significantly higher intensity of CD235a on CECs versus RBCs, which enables CECs to become efficient HIV carriers. Our observations suggest cluster formation of CD235a on CECs following interactions with HIV-1, which was significantly abrogated in the presence of anti-CD235a blocking antibody. It has been suggested that RBC glycoproteins (e.g., CD235a) may act as decoy receptors for other pathogens (61). In this context, the better binding of HIV to CD235a on CECs than RBCs may constitute an advantage for the virus to get a free and more successful ride by CECs to different anatomical locations.

To better correlate the *in vitro* findings with our *in vivo* observations in HIV-infected individuals, we further studied CECs in patients. In agreement with CECs isolated from the cord blood and placental tissues, CECs from HIV-infected individuals also express substantial levels of DARC and CR1 and subsequently enhance HIV replication via ROS. Similar results were observed when the frequency and functionality of CECs from anemic individuals were studied.

It appears that CECs, regardless of their origin, have similar immunological properties and adhere to HIV and enhance its infection/replication in CD4^+^ T cells *in vitro*.

Although there is more HIV associated with white blood cells (e.g., CD4 cells, monocytes, and dendritic cells [DCs]), the strikingly greater number of erythrocytes in the blood makes these cells a major source of viral RNA and subsequently transmissible virus (35, 62). If erythrocyte-associated HIV through immune complexes serves as a viral reservoir (35, 56, 62), the greater expression of CR1, DARC, and CD235a on CECs provides additional evidence that CECs might act more efficiently than their older siblings to carry the virus to different target cells within the body. Anemia is commonly reported among HIV-infected children with underlying nutritional deficiencies and endemic parasitic infections, such as malaria and helminth infections, which lead to RBC destruction, or decreased production. Therefore, these conditions lead to an abundance of CECs in the periphery (37). Subsequently, the presence of CECs may enhance HIV replication in infected individuals or enhance HIV infection/acquisition under certain circumstances. For instance, the physiological abundance of CECs in the cord blood, placenta, and peripheral blood of mothers during pregnancy may enhance mother-to-child HIV transmission (MTCT). Although in the present study, this hypothesis has not been tested, CECs may play an important role in MTCT. In addition, extramedullary erythropoiesis under pathological conditions (e.g., pregnancy, chronic infections, and cancer) (37) may also impact HIV infection/replication and should be taken into consideration.

Strikingly, the presence of infectious viral particles inside CECs obtained from HIV-infected individuals suggests the potential role of CECs as HIV reservoirs/carriers. HIV transmission to CD4^+^ T cells even after tenofovir and raltegravir treatment implies that most transfer occurs as a result of productive infection of CECs but that some (∼10%) transfer is not infection dependent, suggesting two separate mechanisms of *trans*-infection. This phenomenon has already been described in dendritic cells and Langerhans cells (LCs) (63–66). The first phase of early transfer to CD4^+^ T cells from DCs/LCs cells occurs within 2 h of HIV exposure, and transfer declines rapidly with time. This phase is not infection dependent but occurs as a result of some sort of endocytosis-related mechanism resulting in the virus being taken up into an intracellular compartment, which the virus can then escape from when the DCs/LCs interact with CD4^+^ T cells (63–66). Nevertheless, the rate of transfer declines with time as the virus becomes degraded by the cells. The second phase of transfer occurs after 72 h as a result of newly formed virions budding off from the surface of cells that have become productively infected and can be blocked by ART. A similar scenario might be the case for CECs—possibly via CD235a-mediated entry. Some of CECs as reticulocytes possess nuclei (5%) or remnants of the translational machinery and other organelles, such as ribosomes, mitochondria, and the Golgi apparatus (67). This might explain viral replication in CECs but no RBCs.

We acknowledge several limitations to our study. Although we were able to find a positive correlation between the percentage of CECs in ART-naive HIV patients and their plasma viral load, this was not the case for HIV patients on ART because HIV-infected individuals on ART have an extremely low to undetectable viral load. Future studies targeting ART-naive individuals will provide better insight into the role of CECs in HIV-1 replication/transmission. Second, the lack of access to ART-naive and HIV-1-infected pregnant mothers prevented us from concluding if the frequency or functionality of CECs in the cord blood/placenta and mother’s blood impacts vertical HIV-1 transmission. Future studies will also be needed to determine whether there is an association between the viral reservoirs in anemic individuals with high percentages of CECs versus nonanemic HIV-1-infected individuals.

In summary, our results support a conceptual model that the interplay between extramedullary erythropoiesis and an abundance of CECs in the periphery may influence HIV pathogenesis. More importantly, these findings provide a novel role for CEC-mediated HIV infection/replication by ROS. By extension, the role of the cell surface receptor CD235a in HIV *trans*-infection by CECs in the presence of ART could highlight the current limitations of ART and present a future target for therapy.

## MATERIALS AND METHODS

### Human subjects.

A total of 90 cord blood and placental tissue samples were obtained from full-term healthy deliveries for these studies and in some cases from subjects with inflammatory bowel disease (IBD). Blood samples from >50 HIV^+^ individuals, 40 healthy controls, and 6 anemic individuals were also obtained. Plasma viral load and clinical data were obtained from the HIV clinic (S.H.).

### Cell isolation.

Peripheral blood mononuclear cells (PBMCs) or cord blood mononuclear cells (CBMCs) were isolated by density gradient using Ficoll-Paque Premium according to our previous reports (14, 68). Placental tissues were processed according to our reports elsewhere (12, 14). CECs were purified from CBMCs, placental tissues, or PBMCs according our previous reports (6, 17). RBCs were isolated from the cord blood by using biotinylated CD235a antibody (Thermo Fisher Scientific). CD4^+^ T cells were also isolated by negative selection using an enrichment kit from Stem Cell Technologies according to manufacturer’s instruction manual. The purity of isolated cells was normally >95% (Fig. S4A and B).

### HIV-1 viral isolates.

The CXCR4-utilizing isolate LAI, the primary CCR5-utilizing HIV-1 strain JR-CSF, and antiretroviral drugs (tenofovir and raltegravir) were obtained from the AIDS Research and Reagent Program at the NIH. The HIV-1 CCR5-tropic GFP-marked virus used to infect primary CD4^+^ T cells (pNL4.3 ADA GFP) (69) was kindly provided by Christopher Power’s laboratory at the University of Alberta.

### *Ex vivo* HIV infection.

Purified CD4^+^ T cells were infected with the LAI, JR-CSF, or enhanced green fluorescent protein (eGFP) viral isolates at multiplicity of infection (MOI) of 0.1 using magnetofection, as we have previously described elsewhere (25, 70). In some experiments, freshly isolated CD4^+^ T cells without prior activation were infected with the virus or isolated CD4^+^ T cells were first cocultured with CECs for 24 h and then infected with the virus.

A fixed number of infected CD4^+^ T cells (normally 10^6^/ml) were cocultured with different ratios of CECs in the presence/absence of l-arginine (2 mM), TGF-β inhibitor (10 μM), *N*-acetyl cysteine (NAC [1 mM]), and apocynin (1 μM to 1 mM) in 24- or 48-well plates for 4 days. In some experiments, CECs and CD4^+^ T cells were cocultured in a Transwell system. CECs are very sensitive to the permeability buffer, and therefore they were lysed when CD4^+^ T cells cocultured with CECs were subjected to the Cytofix/Cytoperm buffer prior to p24 intracellular staining. A LIVE/DEAD Fixable Aqua Dead cell stain kit (Thermo Fisher Scientific) was used to exclude dead cells. As shown in Fig. S1C, no significant difference in the viability of CD4^+^ T cells in the presence or absence of CECs was observed.

### Flow cytometry analysis.

Cells were stained with anti-CD4 (clone RPA-T4) and anti-CD3 (clone SK7) conjugated antibodies (BD Bioscience). Cells were then fixed and permeabilized with Cytofix/Cytoperm (BD Bioscience) followed by intracellular staining with phycoerythrin (PE)-conjugated KC57 anti-p24 antibody (Beckman Coulter).

CR-1 (E11), DARC (2C3), CD71 (OX-26), CD235a (GA-R2), CCR5 (3A9), and CXCR4 (12G5) all were purchased from BD. ROS staining (Sigma) and staining with the MitoSOX red mitochondrial superoxide indicator (ThermoFisher Scientific) were performed per the manufacturers’ protocols. Paraformaldehyde-fixed cells were acquired using a BD LSR Fortessa flow cytometer (BD Biosciences) and analyzed with FlowJo (version 10) software.

### *trans*-infection assays.

Enriched CECs were incubated with HIV at MOI of 0.1, either with or without magnetofection, as described previously. Then cells were washed extensively and cocultured with either PHA-activated or inactivated CD4^+^ T cells. In some experiments, CECs or RBCs were preincubated with human recombinant CCL5 (1 to 100 ng/ml [R&D]), CD35 blocking antibody (5 to 20 μg/ml, clone J3D3 [Beckman Coulter]), anti-CD235a blocking antibody (2.5 to 10 μg/ml, clone 6A7M [Bio X Cell]) or its isotype IgG1 control (2.5 to 10 μg/ml, MOPC-21 [Bio X Cell]) for an hour prior to infection. CECs (2 × 10^6^) from HIV-infected individuals were lysed in RBC lysis buffer (200 μl for 2 min) followed by coculture with autologous CD4^+^ T cells (1 × 10^6^). RBC lysis buffer was made of ammonium chloride (NH_4_CL, 8.29 g), potassium bicarbonate (KHCO_3_, 1 g) and EDTA (0.037 g) in 1 liter of H_2_O (pH 7.4) (17).

### Phagocytosis assay.

Bordetella pertussis bacteria were labeled with CFSE (carboxyfluorescein succinimidyl ester) for 8 min. CFSE-labeled B. pertussis bacteria (1 × 10^6^) were incubated with 1 × 10^6^ CBMCs in 200 μl of culture medium (RPMI [Sigma]) plus 10% fetal bovine serum (FBS) for 1 h at 37°C. In some experiments, CECs were removed or cultures were supplemented with l-arginine (1 mM). Cells were washed extensively with phosphate-buffered saline (PBS) prior to staining with anti-CD11b cells and analyzed by ImageStream.

### Gene expression analysis.

RNA extraction gene expression assays were performed according to our previous reports (12, 25, 70). Prior to RNA extraction, CD4^+^ T cells were repurified following coculture with CECs in order to prevent any RNA contamination from CECs. To determine the expression levels of genes involved in ROS production, SYBR green master mix (Qiagen) was used targeting the NOX1 (QT00025585), NOX2 (CYBB, QT00029533), NOX3 (QT00044737), NOX4 (QT00057498), NOX5 (QT00021924), DUOX1 (QT00038346), and DUOX2 (QT00012236) genes. TaqMan probes were used to detect expression levels of NF-κB (NFKBIA; Hs00355671-g1) and arginase-2 (Arg2; Hs00982833-ml). The Beta-2 microglobulin (B2M, QT00088935) and β-actin (ACTB, Hs01060665_g1) genes for Qiagen and TaqMan chemistry, respectively, were used as the reference genes. Each sample was run in duplicate, and individual reaction mixtures contained 10 ng cDNA in a total reaction volume of 20 μl. The gene expression of the targeted genes was calculated by the 2^−ΔΔ^*^CT^* (threshold cycle) method, and specific mRNA levels were expressed as fold change over the uninfected condition.

### RNA sequencing.

RNA libraries were constructed using the TruSeq RNA Library Prep kit v2 (Illumina) and then sequenced on a NextSeq 500 instrument (Illumina) with a 150-bp paired-end protocol at an average depth of ∼23.3 million reads per sample (The Applied Genomic Core), University of Alberta.

FASTQ files were subjected to quality control trimming of all bases with a Q score lower than 20, and trimmed reads with length shorter than 75 bp were discarded. Pseudo-alignments were conducted with Kallisto, with 100 permutations, to generate abundance estimates (counts). Raw counts from the sample reads were subjected to statistical analysis using the R package EdgeR (version 3.20.9). Raw counts were transformed into a list of differentially expressed genes (DEG) and subjected to differential gene expression analysis. Significantly upregulated and downregulated genes were identified as having a false-discovery rate (FDR) of <0.05.

For heat mapping analysis, significantly differentially expressed genes with a log fold change (FC) of <−2 and log FC of >2 and with a count per million (CPM) in at least two samples of >2 were used. The genes were plotted with the package pheatmap using Euclidean clustering and Ward aggregation.

Clusters identified by heat mapping were subjected to Gene Ontology analysis using the Biological Process Gene Ontology function on the Gene Ontology Consortium website.

### ImageStream analysis.

An Amnis ImageStream Mark II (EMD Millipore) was used to collect at least 5,000 images for each sample and each condition. Analysis was performed by selecting fluorescence intensity for each targeted cell marker.

### Statistical analysis.

Statistical comparison between various groups was performed by the *t* test using PRISM software. Also, differences were evaluated using one-way analysis of variance (ANOVA) followed by Tukey’s test for multiple comparisons. Results are expressed as mean ± standard error of the mean (SEM). *P* < 0.05 was considered statistically significance.

### Study approval.

The appropriate institutional review boards at the University of Alberta approved the studies. All study participants gave written informed consent prior to participate in this study (protocol no. Pro00056685, Pro00046080, and Pro00046064). All study participants were adults >20 years old.

### Data availability.

Raw data have been deposited in the SRA database of NCBI and are publicly available under accession no. PRJNA529907 at https://www.ncbi.nlm.nih.gov/sra.
