# Supplementary figures and images for: CD71+ Erythroid Cells Exacerbate HIV-1 Susceptibility, Mediate trans-Infection, and Harbor Infective Viral Particles
Source: mBio. 2019 Nov 26;10(6):e02767-19. doi: 10.1128/mBio.02767-19 (PMC6879723; doi:10.1128/mBio.02767-19)

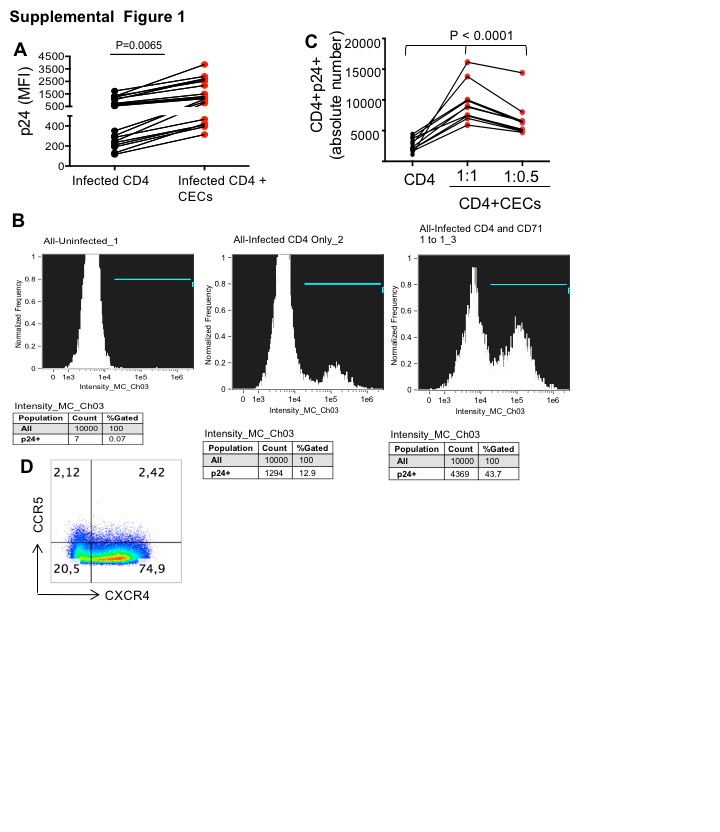

Supplement: FIG S1 [file mBio.02767-19-sf001.jpg]

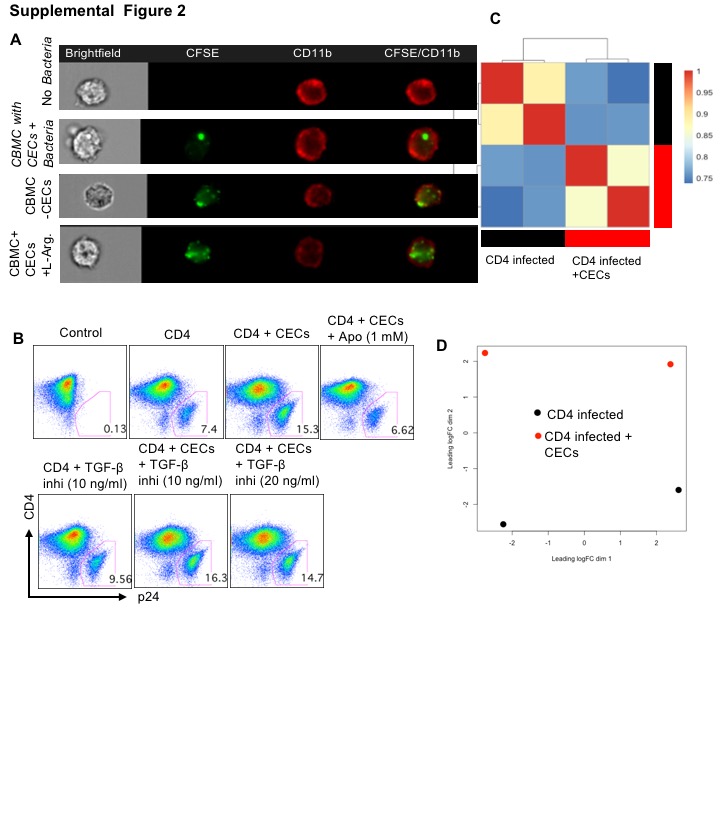

Supplement: FIG S2 [file mBio.02767-19-sf002.jpg]

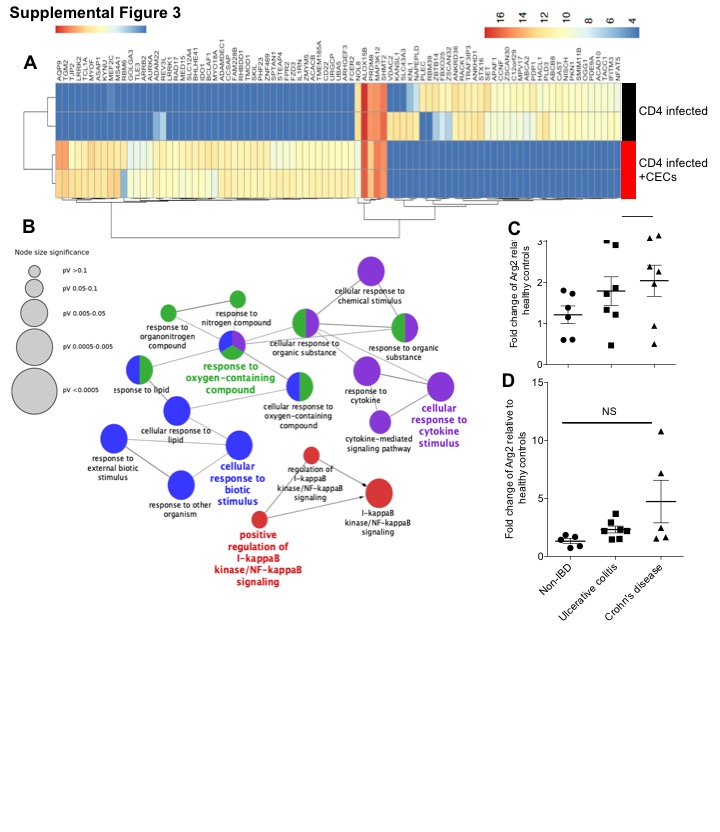

Supplement: FIG S3 [file mBio.02767-19-sf003.jpg]

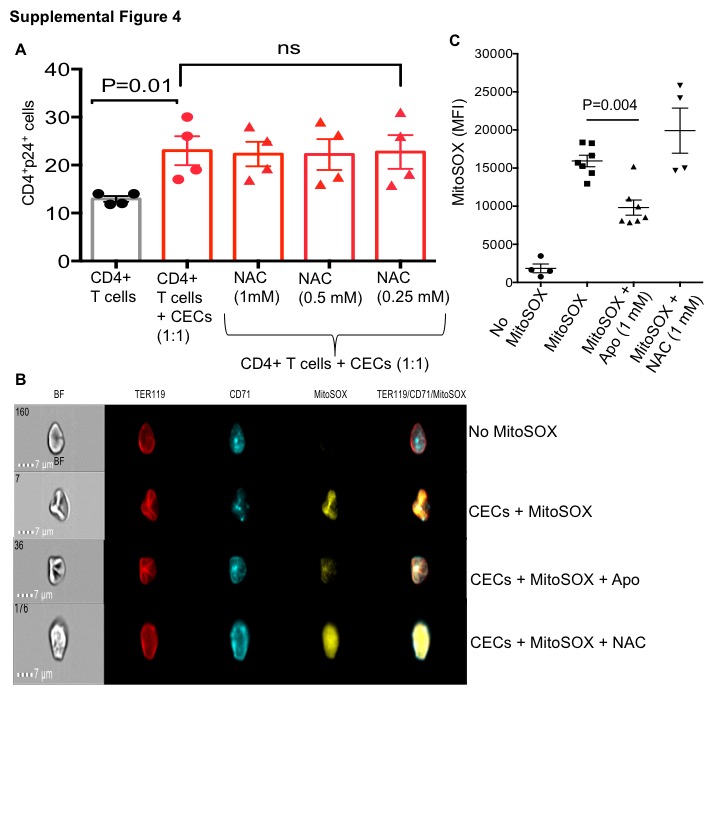

Supplement: FIG S4 [file mBio.02767-19-sf004.jpg]

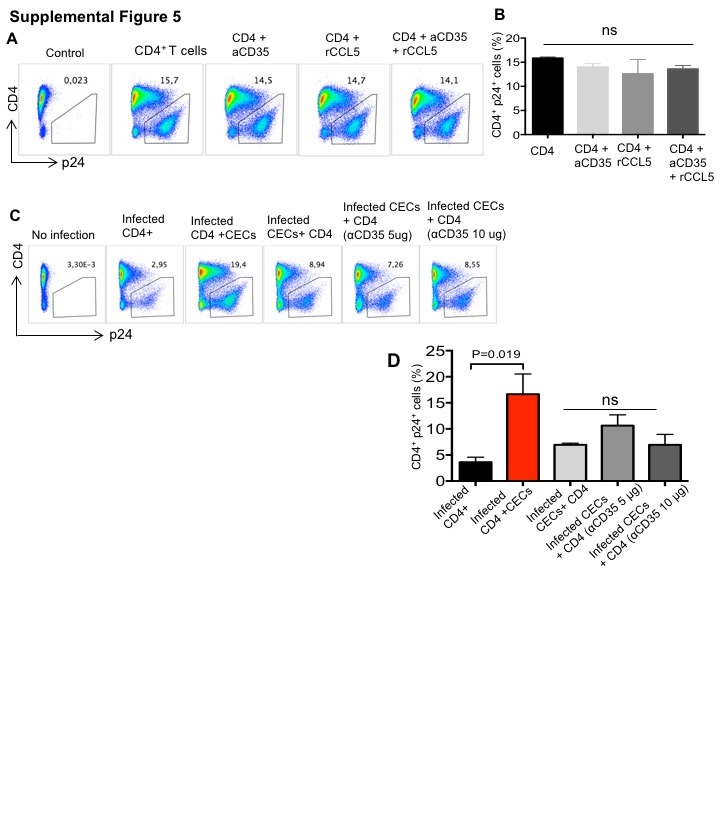

Supplement: FIG S5 [file mBio.02767-19-sf005.jpg]

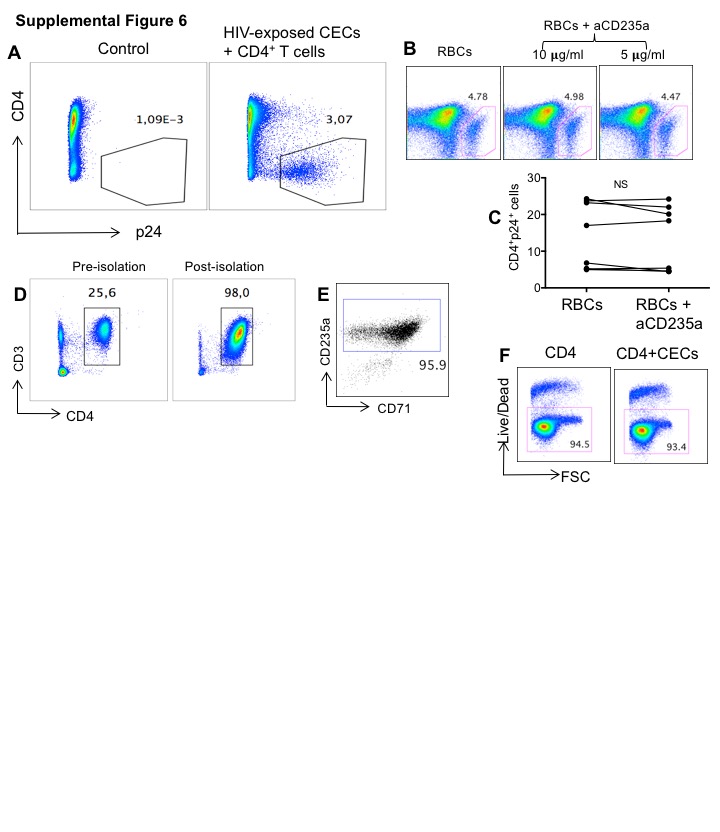

Supplement: FIG S6 [file mBio.02767-19-sf006.jpg]
